# Computational Evaluation of Necrostatin-1 Analogues as Potential RIPK1 Inhibitors

**DOI:** 10.3390/ijms27219616

**Published:** 2026-10-27

**Authors:** Errikos Petsas, Christos T. Chasapis

**Affiliations:** Laboratory of Organic Chemistry, Department of Chemistry, National and Kapodistrian University of Athens, 15771 Athens, Greece; errpets@chem.uoa.gr

**Keywords:** RIPK1 kinase, necroptosis, inflammation, Necrostatin-1, induced fit docking, Desmond molecular dynamics, SwissADME, medicinal chemistry

## Abstract

Receptor-interacting serine/threonine-protein kinase 1 (RIPK1) regulates necroptotic cell death and sterile inflammation, representing an established therapeutic target for degenerative and inflammatory disorders. Here, three putative hydantoin-based analogues bearing distinctive N-substitutions—an ethylcyclobutyl derivative (Compound **1**), an ethoxyphenylethyl analogue (Compound **2**), and a 5-chloroindole (3-fluoro-4-methylbenzyl) derivative (Compound **3**)—were investigated against the prototypical inhibitor Necrostatin-1 using virtual screening, induced-fit docking, 500 ns molecular dynamics simulations and pharmacokinetic profiling. Induced Fit Docking within the RIPK1 kinase domain indicated that Compound **3** (−9.1 kcal/mol) and Compound **1** (−7.9 kcal/mol) achieved favorable predicted binding scores surpassing Necrostatin-1 (−7.3 kcal/mol), whereas Compound **2** displayed a considerably weaker binding score (−4.1 kcal/mol). All-atom molecular dynamics simulations revealed that Compound **1** maintained high dynamic stability with ligand RMSD constrained to 1.2–2.0 Å and sustained hydrogen bonding with Leu157. Conversely, Compound **2** dissociated from the primary binding pocket, while Compound **3** deviated from its initial docking pose (RMSD 7.0–9.2 Å) to engage a secondary interaction network with Asn170, Glu171 and Lys45. In silico ADME profiling predicted that all three candidates comply with Lipinski and Veber drug-likeness criteria with high predicted intestinal absorption. These computational findings provide putative structural insights into kinase-ligand interactions, prioritizing candidate molecules for chemical synthesis and in vitro biological evaluation.

## 1. Introduction

Receptor-Interacting Serine/Threonine-Protein Kinase 1 (RIPK1) [1] is a key regulator of cell fate, operating downstream of death receptors such as TNFR1 to direct cell survival, apoptosis, or necroptosis [2,3,4]. While its scaffolding properties are required for NF-κB activation and cell survival, its enzymatic kinase activity is essential for the execution of necroptotic cell death [5,6]. Necroptosis is a regulated form of necrotic cell death defined by early plasma membrane rupture and the subsequent release of damage-associated molecular patterns (DAMPs), which drive severe local and systemic inflammatory responses [7,8].

Pathological overactivation of RIPK1 kinase activity and unrestrained necroptosis are established contributors to tissue injury across acute and chronic inflammatory pathologies [9,10]. In particular, excessive RIPK1-dependent cell death plays a documented role in ischemic reperfusion injury of the brain (ischemic stroke) and myocardium (myocardial infarction) [11,12], neurodegenerative conditions including Alzheimer’s disease and amyotrophic lateral sclerosis, and chronic systemic inflammation such as inflammatory bowel disease and acute kidney injury [13,14]. Selective small-molecule inhibition of the RIPK1 kinase domain therefore represents a rational therapeutic strategy aimed at preventing necrotic tissue damage and reducing the secondary sterile inflammation associated with these diseases, without perturbing the non-enzymatic scaffolding functions required for basal immune homeostasis [15,16].

The identification of Necrostatin-1 (Nec-1) as a prototype allosteric inhibitor established the hydantoin-indole scaffold as a validated starting point for targeting RIPK1 [17,18]. Necrostatin-1 binds the inactive kinase conformation, occupying a distinct lipophilic pocket adjacent to the ATP-binding cleft as characterized crystallographically (PDB ID: 4ITH) [19]. However, Necrostatin-1 exhibits well-documented pharmacokinetic and translational liabilities, notably rapid in vivo metabolic clearance, short plasma half-life, and metabolic instability largely attributable to its metabolically labile thiocarbonyl group (C=S) and susceptibility to oxidative desulfuration [20,21]. These limitations warrant structural modifications around the hydantoin scaffold aimed at optimizing steric fitting, reinforcing anchoring contacts within the allosteric pocket, and replacing labile motifs to enhance pharmacokinetic endurance and oral bioavailability [22,23]. In this context, exploring diverse aliphatic and aromatic N-substitutions—such as constrained cycloalkyl rings or substituted benzyl moieties—presents a rational chemical strategy to probe pocket complementarity while addressing metabolic vulnerabilities. High-resolution structural data of the RIPK1 catalytic domain provide a reliable basis for investigating how specific substitutions on this core alter binding and complex dynamics [22,23].

In this study, an in silico workflow was applied to evaluate three putative analogues of Necrostatin-1 selected via the SwissSimilarity platform from the Enamine screening library. The initial binding geometries and interaction networks within the catalytic domain of RIPK1 (PDB ID: 4ITH) were modeled using Induced Fit Docking and two-dimensional interaction analysis. Long-timescale 500 ns molecular dynamics simulations were then conducted in explicit solvent to examine complex stability, root-mean-square deviations, temporal persistence of protein–ligand contacts, and dynamic conformational evolution via 3D snapshot superimpositions. Finally, in silico physicochemical and pharmacokinetic profiling was performed using SwissADME to evaluate the predicted physicochemical parameters and drug-likeness profiles of the prioritized candidate molecules.

## 2. Results

### 2.1. Induced Fit Docking Analysis

The binding affinities and conformational orientations of the investigated chemical entities within the kinase domain of RIPK1 (PDB ID: 4ITH) are analyzed. Using Necrostatin-1 as the reference inhibitor, Induced Fit Docking simulations were performed to account for receptor flexibility and provide quantitative energetic ranking of each ligand.

The binding affinities of the selected compounds and the reference inhibitor Necrostatin-1 were analyzed within the kinase domain of RIPK1 (PDB ID: 4ITH) using the Induced Fit Docking protocol. The calculated docking scores and structural descriptions are presented in Table 1.

Compound **3** demonstrated the most favorable binding score (−9.081 kcal/mol), exceeding that of the reference compound Necrostatin-1 (−7.337 kcal/mol). Compound **1** exhibited a comparable docking score of −7.793 kcal/mol, indicating that the ethylcyclobutyl moiety is accommodated within the lipophilic subpocket of the binding site without steric clashes. In contrast, Compound **2** yielded a weaker docking score of −4.157 kcal/mol, reflecting unfavorable steric hindrance upon accommodating the elongated ethoxyphenylethyl substituent within the catalytic pocket.

### 2.2. Two-Dimensional Ligand-Protein Interaction Profiles

Two-dimensional ligand-protein interaction profiles detail the specific intermolecular interaction networks formed between the docked ligands and key residues lining the catalytic cleft of RIPK1 (PDB ID: 4ITH). Two-dimensional interaction diagrams are analyzed to map hydrogen bonds, cation-pi interactions and hydrophobic contacts that suggest initial binding modes.

**Figure 1:**
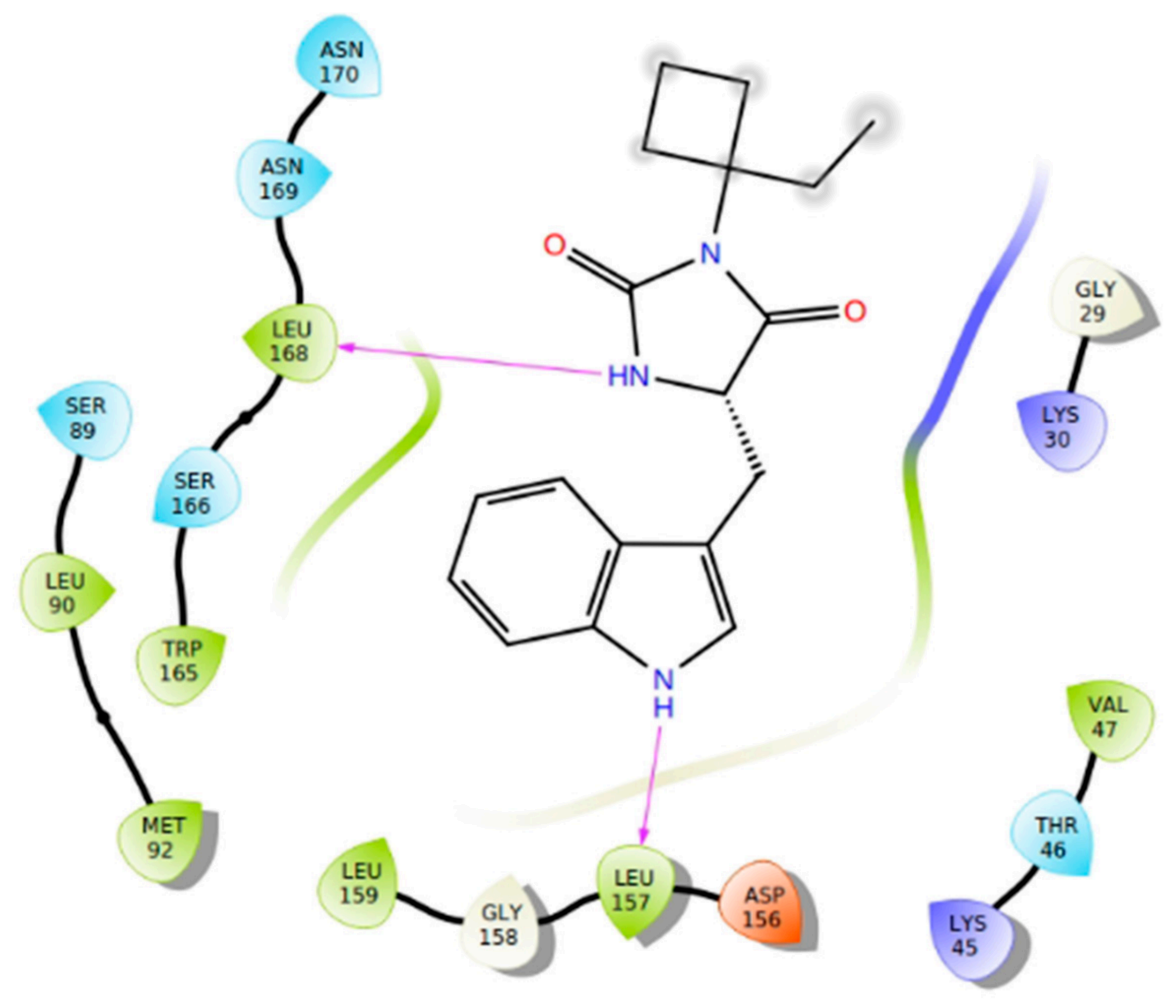
Two-dimensional interaction diagram of Compound **1** docked into the RIPK1 kinase domain (PDB ID: 4ITH), highlighting hydrogen bonds with Leu157 and Leu168 alongside surrounding hydrophobic residues. The diagram was generated using the Ligand Interaction Diagram module in Maestro (Schrödinger, LLC, New York, NY, USA).

The binding pose of Compound **1** within the RIPK1 active site is stabilized by two directional hydrogen bonds, involving the indole NH as a donor toward the backbone carbonyl of Leu157 and the hydantoin NH donating toward the carbonyl oxygen of Leu168. Hydrophobic complementarity is provided by non-polar contacts with Val47, Leu90, Met92, Leu159 and Trp165, which envelop the indole ring and the ethylcyclobutyl substituent without introducing steric strain (Figure 1).

**Figure 2:**
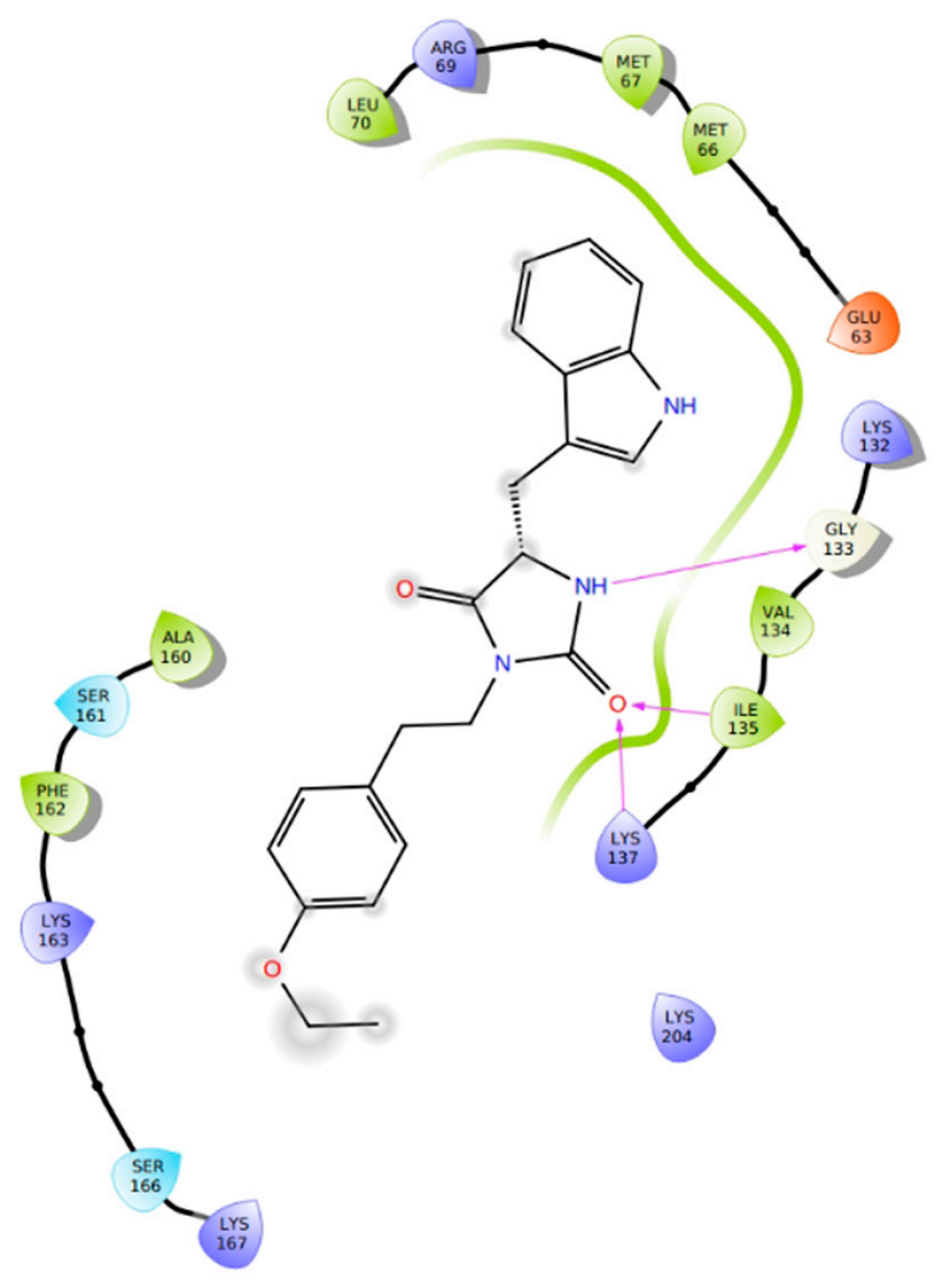
Two-dimensional interaction diagram of Compound **2** in the RIPK1 active site (PDB ID: 4ITH), illustrating polar contacts with Gly133, Ile135, Lys137, and the peripheral positioning of the ethoxyphenylethyl group. The diagram was generated using the Ligand Interaction Diagram module in Maestro (Schrödinger, LLC, New York, NY, USA).

Compound **2** exhibits an altered orientation wherein the hydantoin NH donates a hydrogen bond to the backbone carbonyl of Gly133, while the neighboring carbonyl oxygen accepts contacts from Ile135 and Lys137. The 4-ethoxyphenylethyl substituent projects toward a peripheral solvent-exposed region defined by residues Phe162, Lys163, and Ser166, failing to engage key residues and resulting in reduced binding energetics compared with the other analogues (Figure 2).

**Figure 3:**
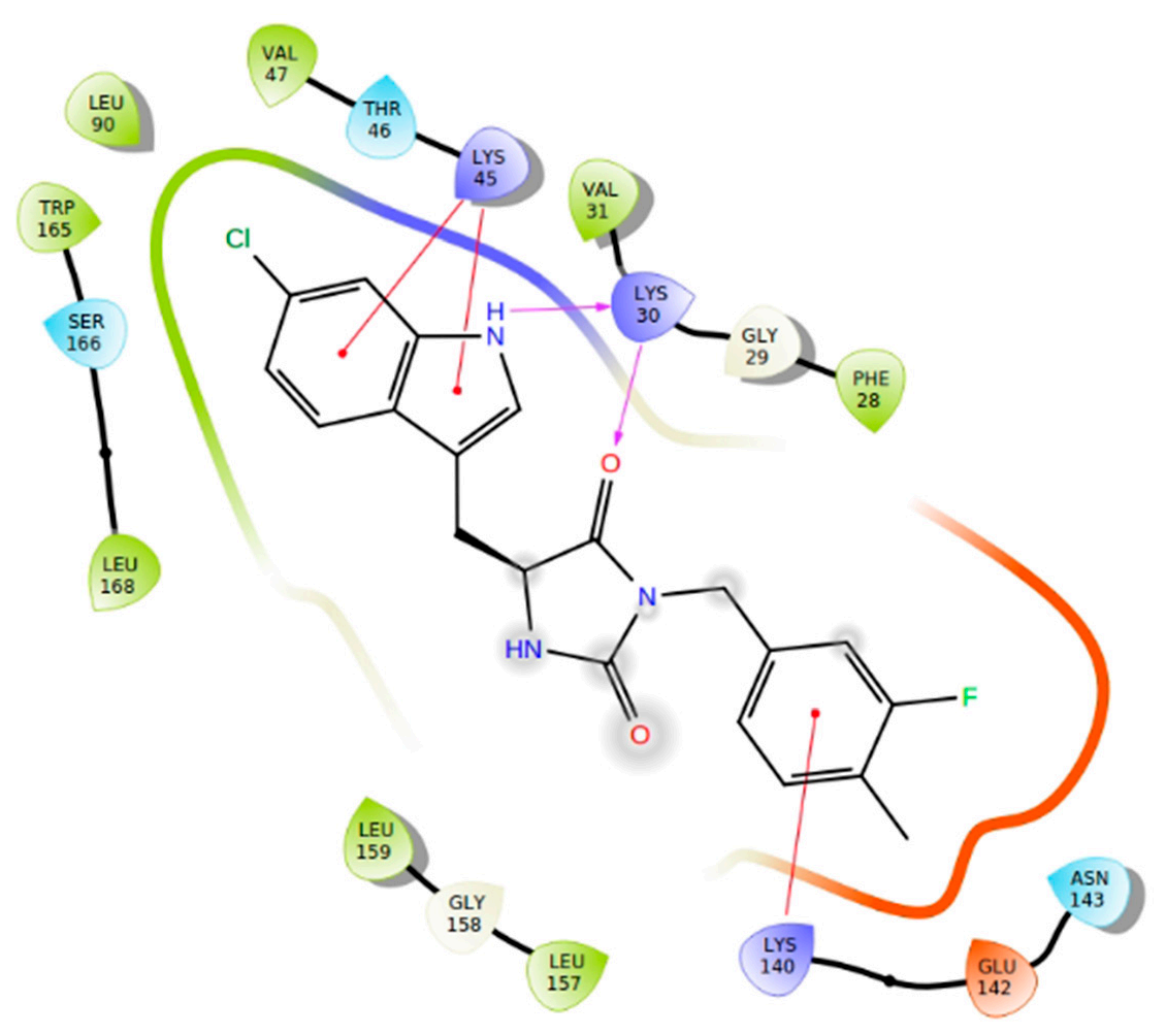
Two-dimensional interaction diagram of Compound **3** bound to RIPK1 (PDB ID: 4ITH), showing the hydrogen-bonding network with Lys30 together with cation-pi interactions mediated by Lys45 and Lys140. The diagram was generated using the Ligand Interaction Diagram module in Maestro (Schrödinger, LLC, New York, NY, USA).

Compound **3** forms a dense contact network, anchored by Lys30 acting concurrently as a hydrogen bond acceptor for the indole NH and as a hydrogen bond donor toward the hydantoin carbonyl oxygen. Additional stabilization is mediated by dual cation-pi interactions between the side chain of Lys45 and both the 5-chloroindole core and the substituted benzyl ring, supplemented by a cation-pi contact between Lys140 and the 3-fluoro-4-methylbenzyl ring (Figure 3).

### 2.3. Molecular Dynamics RMSD Analysis

Here, the dynamic stability and structural evolution of the docked RIPK1-ligand complexes (PDB ID: 4ITH) are examined over 500 ns molecular dynamics trajectories executed via the Desmond engine. Root-mean-square deviation (RMSD) profiles for the protein C-alpha backbone and the ligand fit on the protein are assessed to monitor system convergence and ligand retention within the binding site.

**Figure 4:**
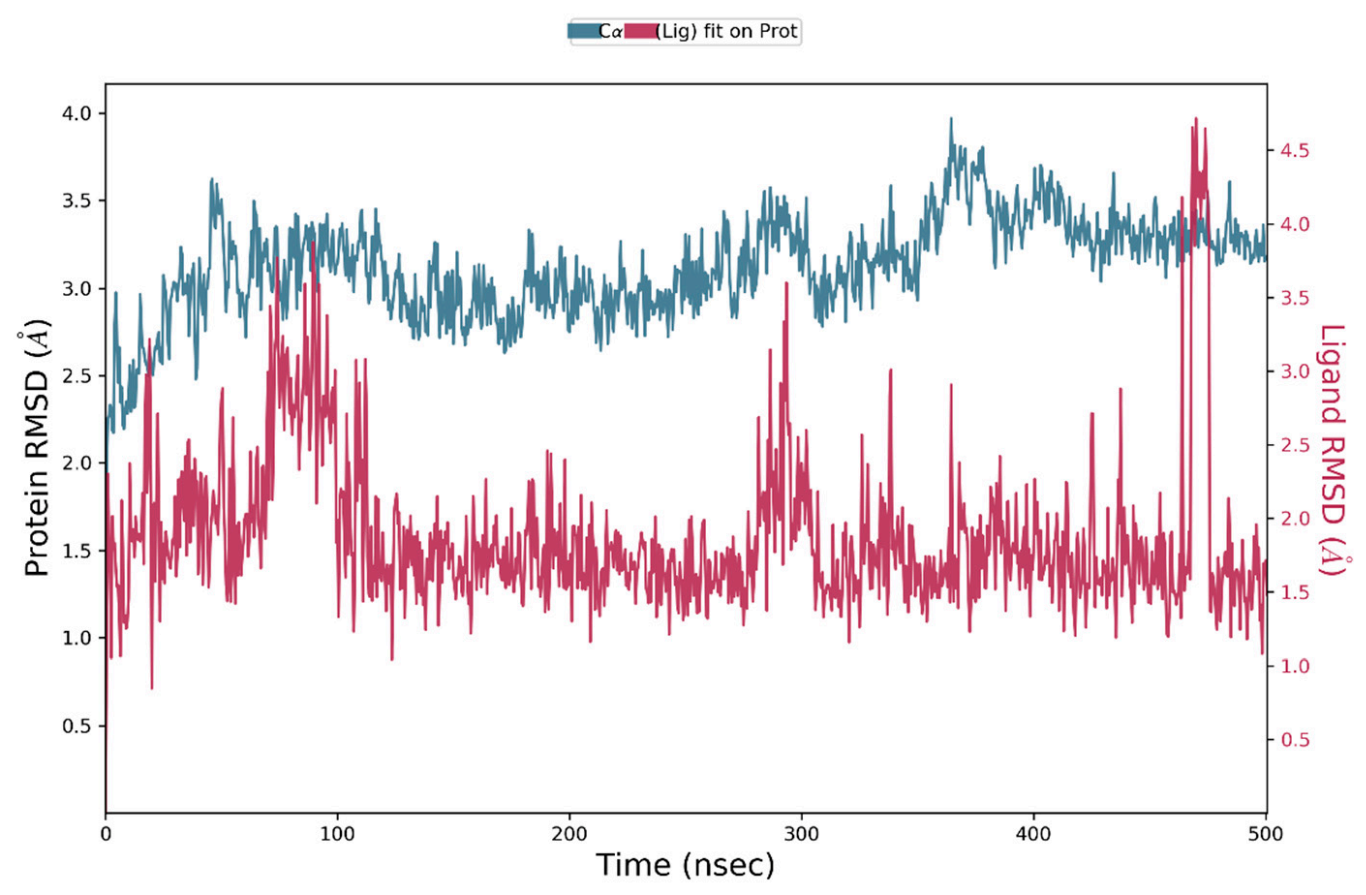
Root-mean-square deviation (RMSD) plot of the protein C-alpha backbone and ligand (fit on protein) for Compound **1** in complex with RIPK1 over a 500 ns Desmond molecular dynamics simulation.

The structural stability of the RIPK1 kinase domain in complex with Compound **1** was examined over a 500 ns molecular dynamics trajectory. The protein C-alpha backbone stabilized rapidly following the initial 50 ns, maintaining consistent fluctuations within 2.7 to 3.4 Å throughout the remainder of the simulation. The ligand RMSD fitted on the protein remained tightly constrained between 1.2 and 2.0 Å, with minor transient fluctuations observed near 100 ns and 470 ns, demonstrating persistent conformational integrity and continuous retention within the binding pocket (Figure 4).

These transient RMSD spikes correspond to temporary rotational adjustments of the solvent-exposed ethylcyclobutyl moiety around its single exocyclic bond, while the core indole scaffold and its pivotal hydrogen bond to Leu157 remained anchored within the deep hydrophobic pocket.

**Figure 5:**
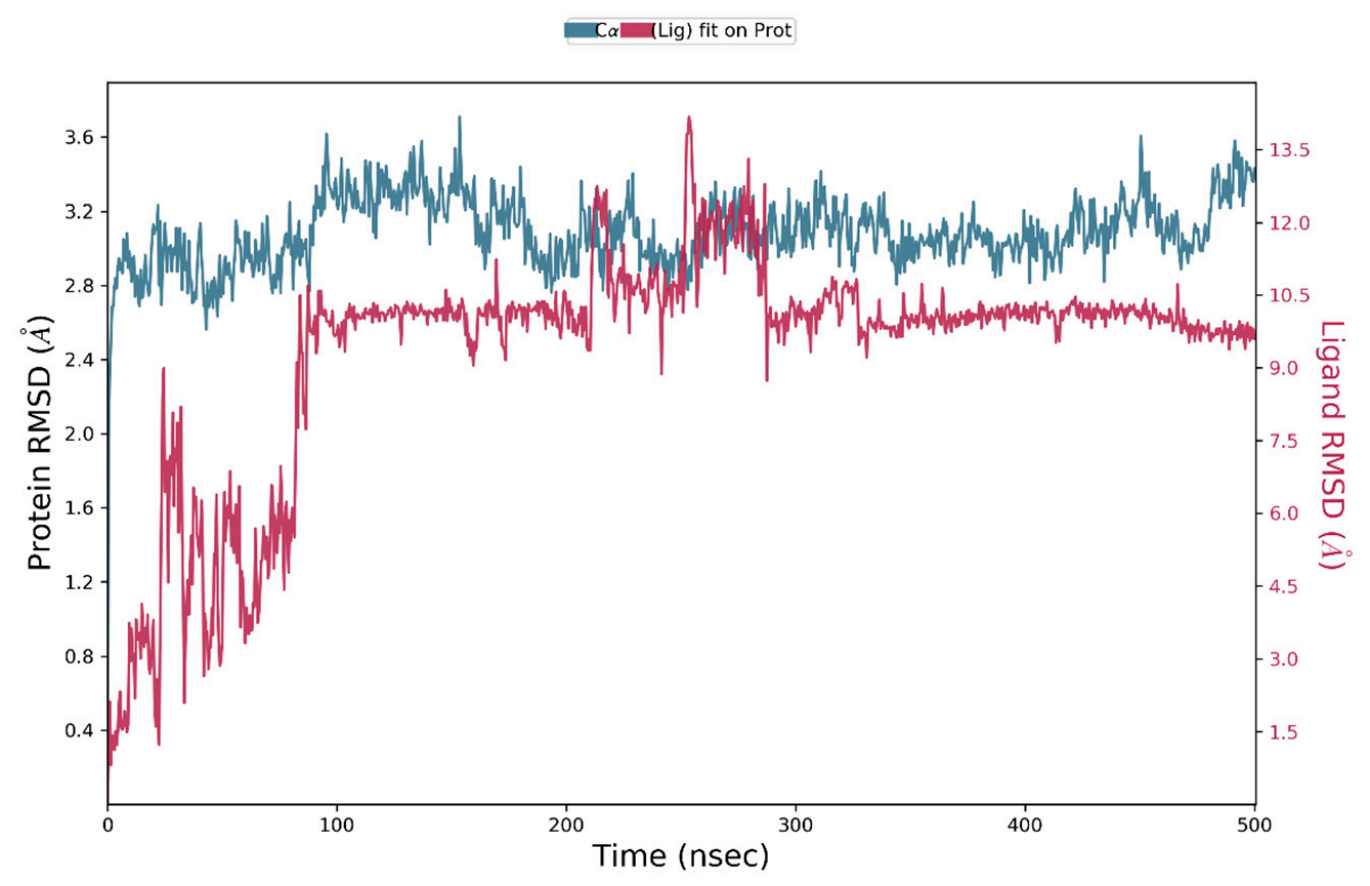
Root-mean-square deviation (RMSD) plot of the protein C-alpha backbone and ligand (fit on protein) for Compound **2** in complex with RIPK1 during the 500 ns trajectory, depicting ligand detachment from the primary binding cleft.

The molecular dynamics trajectory for Compound **2** exhibited stable protein C-alpha RMSD values oscillating between 2.8 and 3.4 Å. In contrast, the ligand RMSD underwent a pronounced conformational displacement at 90 ns, rising abruptly from 4.5 Å to approximately 10.0 Å. The ligand maintained an elevated RMSD profile between 10.0 and 12.0 Å until the completion of the 500 ns run, which reflects disengagement from the primary docking site and stable relocation to an adjacent peripheral pocket on the protein surface (Figure 5).

**Figure 6:**
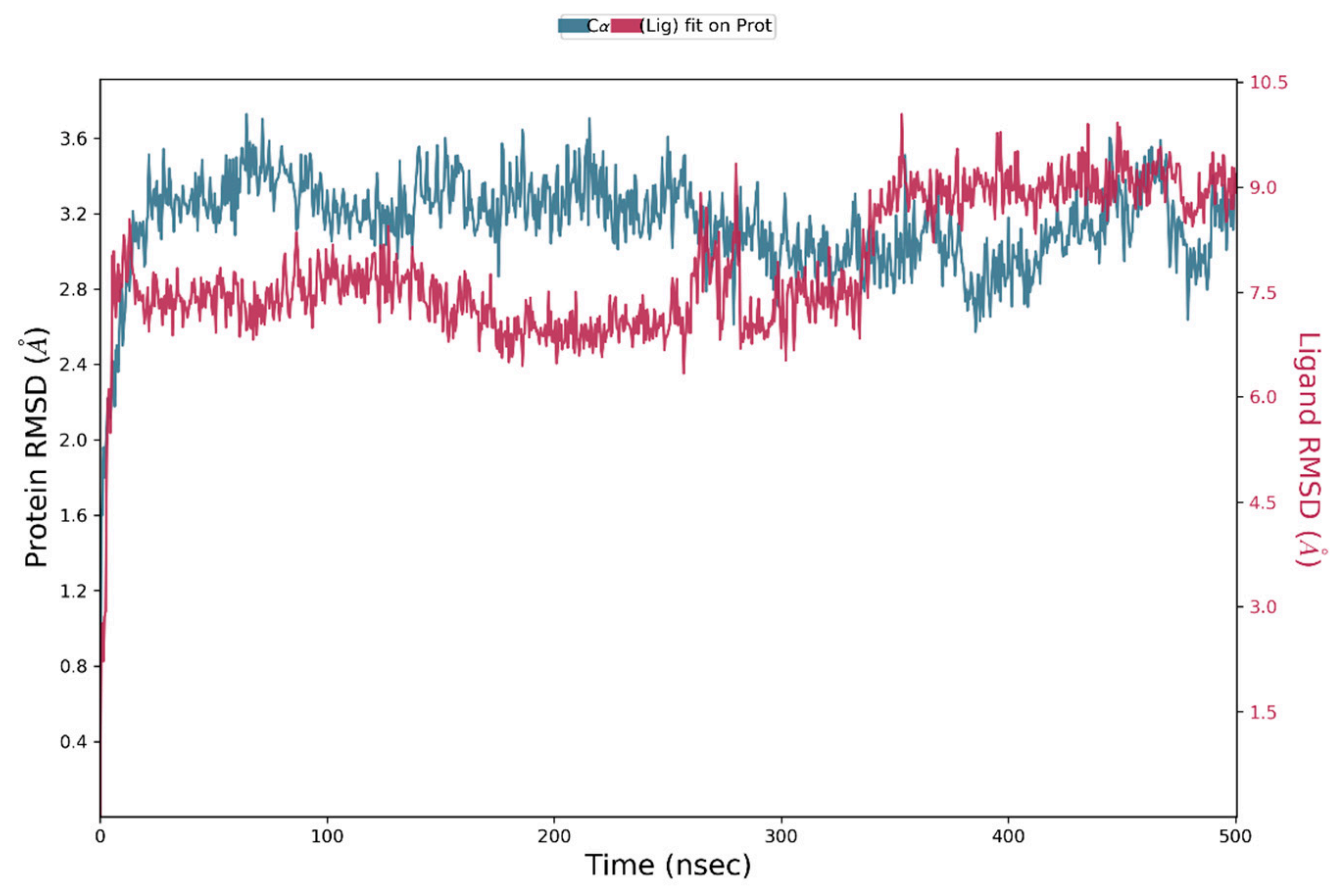
Root-mean-square deviation (RMSD) plot of the protein C-alpha backbone and ligand (fitted to the protein) for Compound **3** in complex with RIPK1 across the 500 ns molecular dynamics trajectory.

Simulation of the Compound **3** complex revealed sustained stability of the protein backbone, with C-alpha RMSD values fluctuating between 2.8 and 3.5 Å across the 500 ns trajectory. However, the ligand RMSD rapidly deviated from the initial docking pose during the first 30 ns, reaching and plateauing within an elevated range of 7.0 to 9.2 Å without further progressive drift. This substantial deviation indicates that the initial static docking pose—and consequently its highly favorable docking score (−9.081 kcal/mol)—was not retained under dynamic solvated conditions. Rather than dissociating into solvent like Compound **2**, Compound **3** underwent a spatial translation within the binding cleft, establishing an alternative, metastable binding orientation stabilized by a distinct local interaction network (Figure 6).

For comparative benchmarking, a 500 ns MD simulation of the reference inhibitor Necrostatin-1 in complex with RIPK1 was also evaluated (Supplementary Materials, Figure S1). Following initial equilibration, the protein C-alpha backbone stabilized at 3.0–3.6 Å, while the ligand RMSD remained consistently plateaued around 5.6–6.0 Å across the entire trajectory without unbinding.

### 2.4. Protein–Ligand Contact Histograms

Protein–Ligand Contact Histograms characterize the temporal persistence and nature of the contacts established between RIPK1 (PDB ID: 4ITH) and each ligand throughout the 500 ns simulation window. Interaction fraction histograms categorize the sustained contributions of hydrogen bonding, water-mediated bridges, and hydrophobic networks on a per-residue basis.

**Figure 7:**
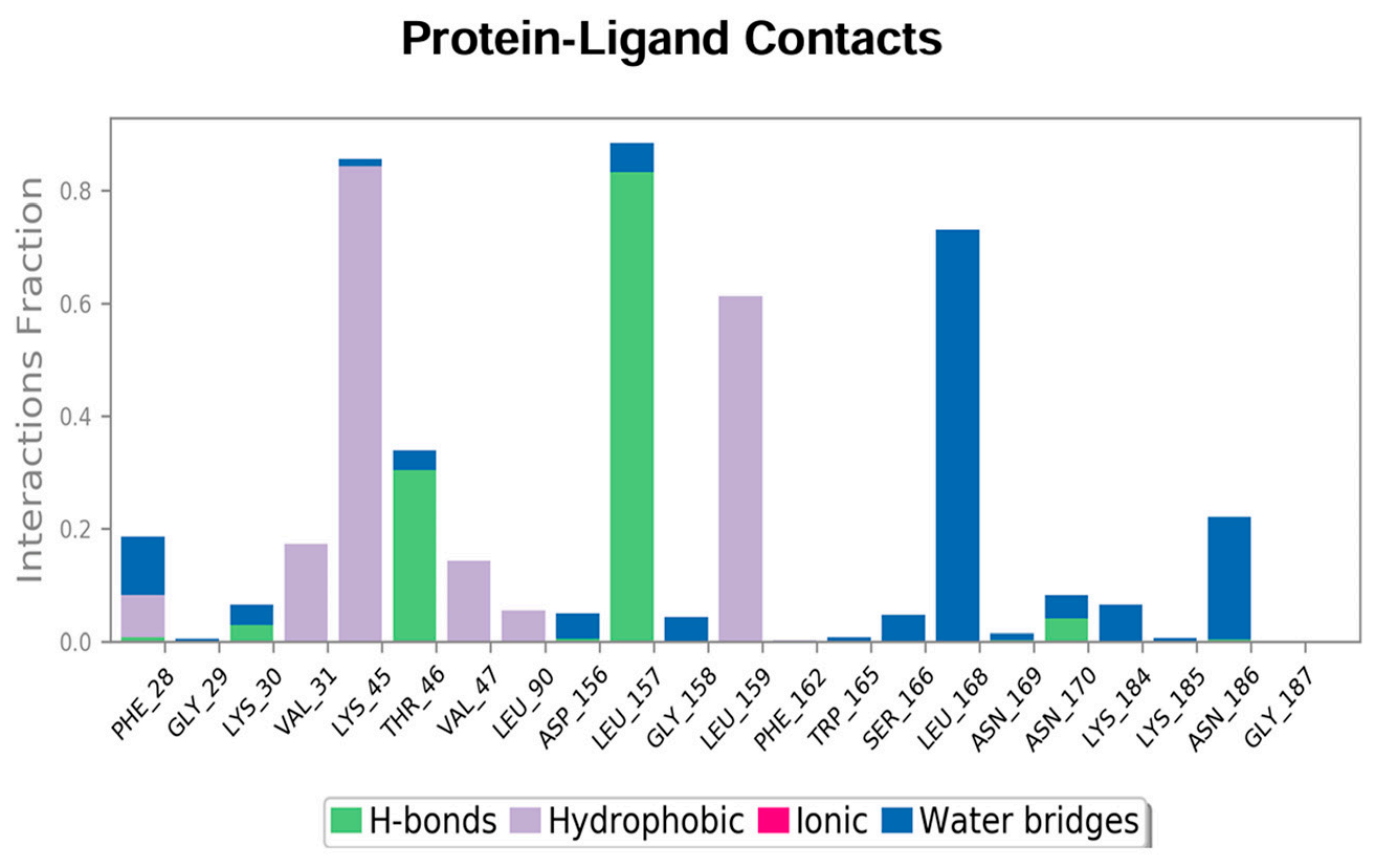
Protein–ligand contacts histogram for Compound **1** across the 500 ns molecular dynamics trajectory, depicting interaction fractions categorized into hydrogen bonds, hydrophobic contacts, and water bridges.

The interaction fraction trajectory for Compound **1** demonstrates that binding persistence is governed by Leu157, which maintains a direct hydrogen bond for over 80% of the simulation window, reinforced by substantial hydrophobic contacts with Lys45 (interaction fraction exceeding 0.8) and Leu159 (approximately 0.6) (Figure 7). Furthermore, Leu168 provides continuous water-mediated bridges for more than 70% of the trajectory, while Thr46 sustains intermittent hydrogen bonds for 30% of the time, thereby establishing a persistent molecular network throughout the active cleft.

**Figure 8:**
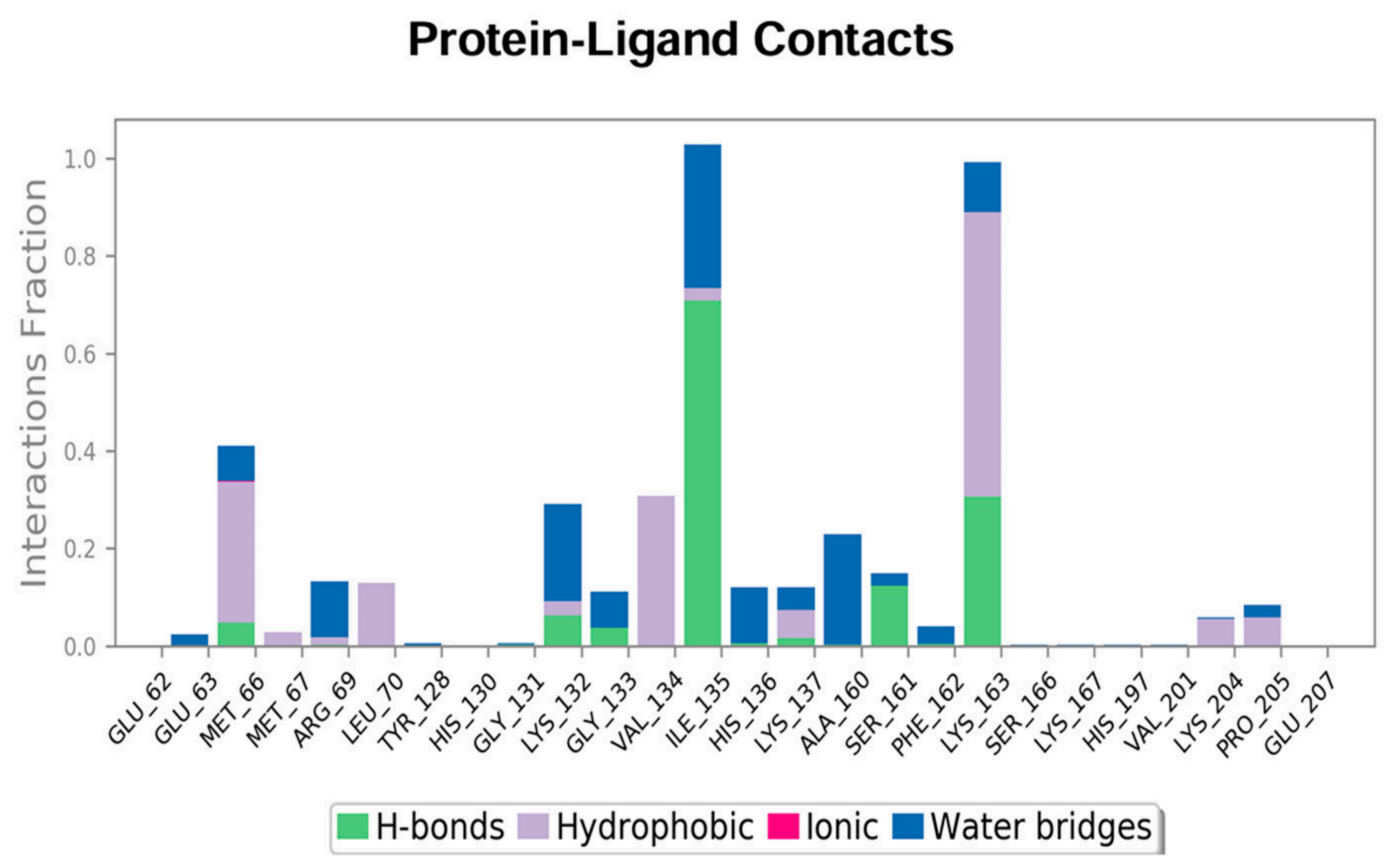
Protein–ligand contacts histogram for Compound **2** across the 500 ns molecular dynamics trajectory, illustrating the sustained interaction fractions observed following ligand relocation.

The contact profile for Compound **2** reflects the structural translation of the ligand to a secondary pocket on the protein surface (Figure 8). In this conformation, interactions are localized at Ile135, which exhibits hydrogen bonding for 70% of the trajectory alongside water bridges for 30% of the run, accompanied by Lys163 contributing hydrophobic contacts (fraction near 0.6) and supplementary hydrogen bonding (fraction near 0.3), while canonical pocket residues, including the DLG motif (Leu157), remain completely disengaged.

**Figure 9:**
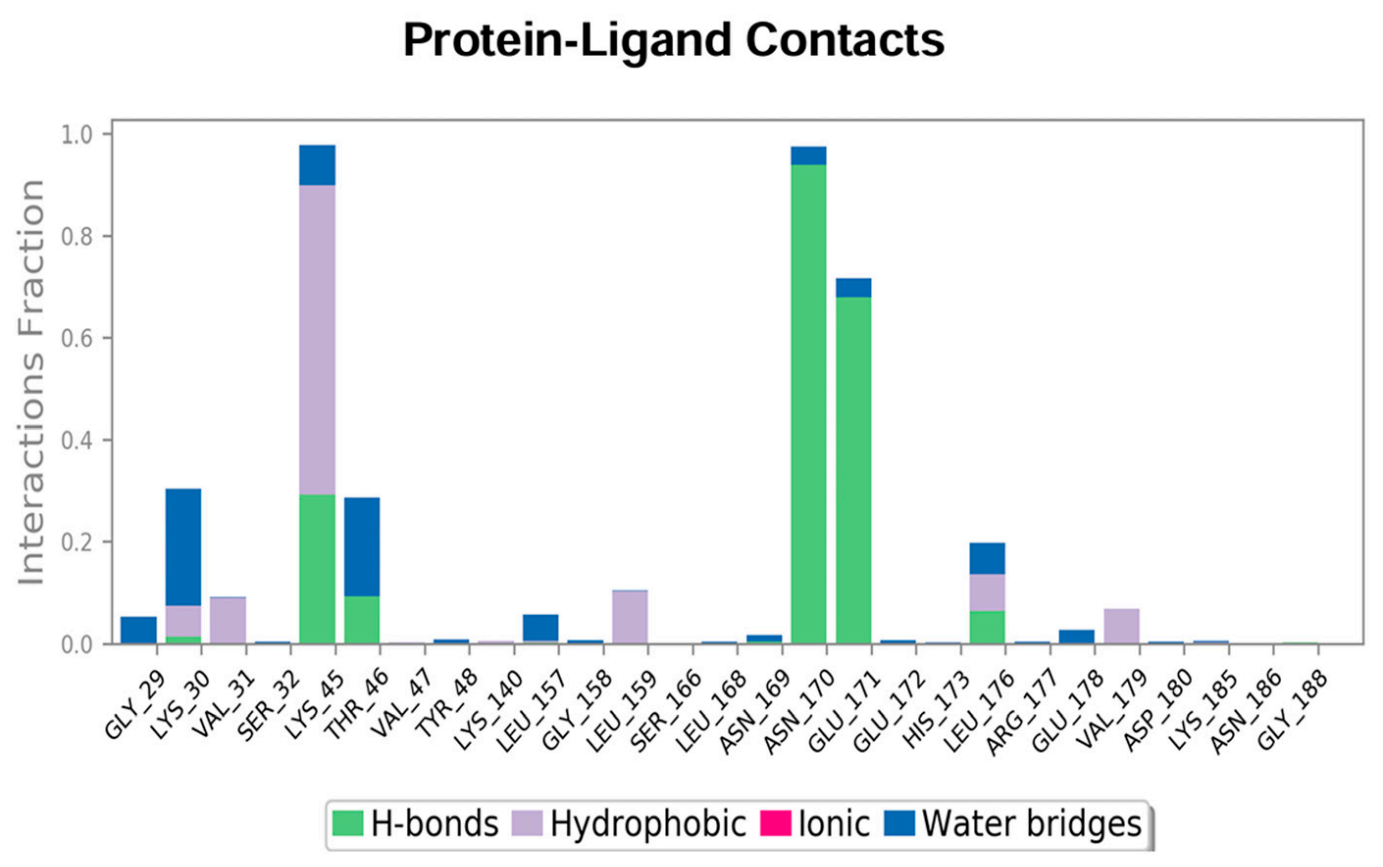
Protein–ligand contacts histogram for Compound **3** across the 500 ns molecular dynamics trajectory, showing the dominant contact fractions across catalytic pocket residues.

For Compound **3**, the contact histogram is defined by primary hydrogen bonding with Asn170 (fraction exceeding 0.9) and Glu171 (fraction near 0.7), which act as the dominant anchoring points during the simulation (Figure 9). Residue Lys45 maintains continuous contact across the simulation (total fraction near 1.0), comprised primarily of hydrophobic interactions with secondary water bridges and polar contributions, while Lys30 and Thr46 further stabilize the complex through intermittent water-mediated and hydrophobic contacts.

The dynamic contact profile of Necrostatin-1 was predominantly anchored by extensive interactions with Lys30 (fraction > 1.0, combining hydrophobic contacts and water bridges) and direct hydrogen bonding with Gly187 (fraction ~0.80), supplemented by Lys45 and Glu171 (Supplementary Materials, Figure S2).

### 2.5. Conformational Superimposition and Comparative Structural Evolution (0 ns vs. 500 ns)

**Figure 10:**
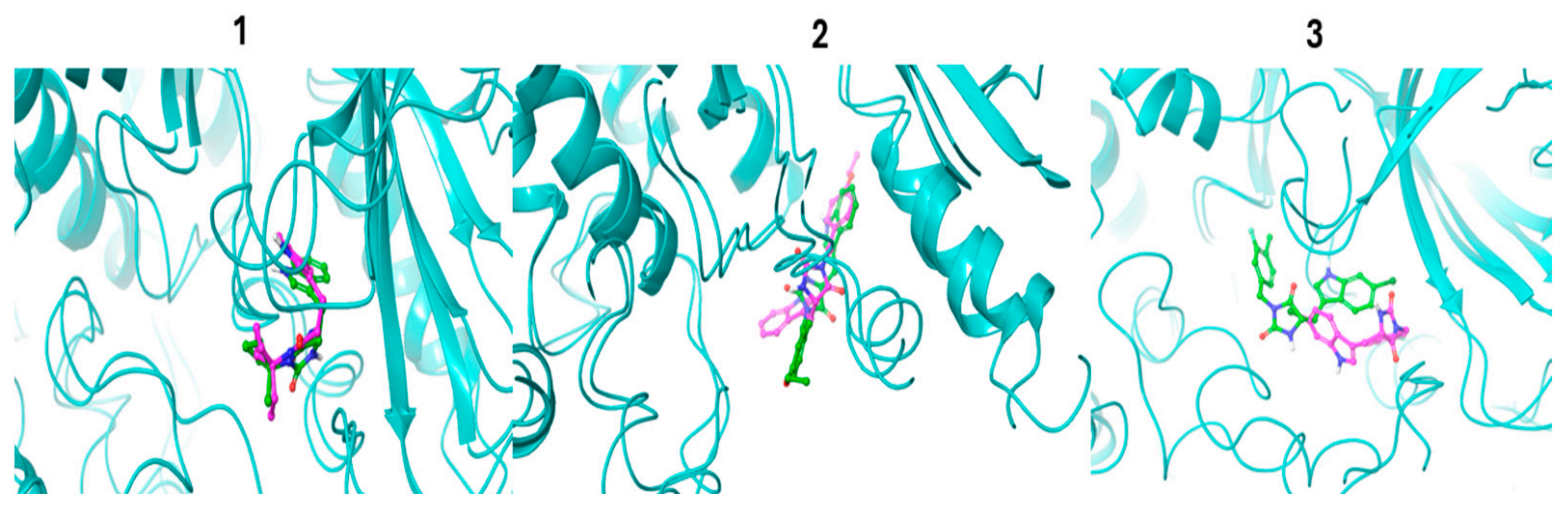
Comparative structural superimpositions of initial (0 ns, green sticks) and final (500 ns, magenta sticks) molecular dynamics snapshots for (**1**) Compound **1**, (**2**) Compound **2** and (**3**) Compound **3** bound within the human RIPK1 kinase domain (PDB ID: 4ITH). The protein backbone is rendered in cyan/teal cartoon ribbon representation. Heteroatoms are color-coded: nitrogen in blue, oxygen in red, chlorine in dark green and fluorine in light green.

To directly visualize the spatial evolution and conformational stability of the investigated chemotypes within the RIPK1 kinase catalytic cleft (PDB ID: 4ITH), structural superimpositions comparing the initial equilibrated snapshot (t = 0 ns) and the final production frame (t = 500 ns) were generated for all three complexes (Figure 10). Compound **1** exhibited remarkable conformational fidelity across the entire 500 ns trajectory. Both the central hydantoin-indole core and the peripheral N-ethylcyclobutyl ring remained tightly aligned with the initial docking coordinates, displaying negligible spatial drift. This persistent orientation directly rationalizes the constrained ligand RMSD (1.2–2.0 Å) and corroborates the sustained hydrogen-bonding network with the hinge/catalytic residue Leu157 and surrounding hydrophobic shielding. In contrast, Compound **2** displayed pronounced conformational divergence. While the upper indole ring partially occupied the lipophilic subpocket, the extended and flexible 4-ethoxyphenylethyl substituent underwent substantial outward rotation and lateral displacement. This conformational rearrangement reflects the steric penalty and high rotational degrees of freedom identified in the docking and contact analyses, highlighting the ligand’s inability to establish stable anchor contacts within the canonical cleft. Furthermore, Compound **3** underwent the most substantial spatial translation within the binding pocket. The initial binding mode was entirely remodeled, with the hydantoin core and the substituted benzyl and chlorinated indole moieties shifting to an adjacent pocket coordinate. This translational adjustment directly mirrors the rapid RMSD displacement (7.0–9.2 Å) observed within the first 30 ns, visualizing the dynamic transition from the initial static pose to the secondary metastable network anchored by Asn170, Glu171, and Lys45.

### 2.6. In Silico Pharmacokinetic and Physicochemical Profiling

The drug-likeness, pharmacokinetic parameters, and physicochemical properties of the reference inhibitor Necrostatin-1 and the examined candidate analogues (Compounds **1**–**3**) are quantitatively assessed using the SwissADME platform. The calculated descriptors, absorption profiles, metabolic inhibition liabilities, and filter compliance metrics are summarized in Table 2, while the corresponding bioavailability radar plots depicting the oral bioavailability space are provided in the Supplementary Materials (Figures S3–S6).

All three compounds exhibit favorable physicochemical properties that strictly comply with Lipinski’s Rule of Five (molecular weight < 500 g/mol, Consensus Log Po/w < 5, H-bond donors <= 5, and H-bond acceptors <= 10) and Veber’s criteria (rotatable bonds <= 10 and TPSA <= 140 Å^2^), yielding an identical bioavailability score of 0.55. The topological polar surface area (TPSA) values remain within an optimal window of 65.20 Å^2^ for Compounds **1** and **3**, and 74.43 Å^2^ for Compound **2**, closely mirroring the reference inhibitor Necrostatin-1 (80.22 Å^2^), consistent with the predicted high gastrointestinal absorption across the entire series. Water solubility calculations via the ESOL topological model classify Compound **1** as soluble (Log S = −3.61), whereas Compounds **2** and **3** are categorized as moderately soluble (Log S = −4.35 and −4.80, respectively), in agreement with the higher aliphatic character of Compound **1** (Fraction Csp3 = 0.44) and balanced lipophilicity (Consensus Log Po/w = 2.52), while Necrostatin-1 is categorized as soluble (Log S = −2.86).

In terms of pharmacokinetic distribution, Compound **1** is predicted to act as a substrate for P-glycoprotein (P-gp), whereas Compounds **2** and **3**, alongside Necrostatin-1, are classified as non-substrates. Blood–brain barrier (BBB) permeation is predicted for all three candidate analogues (Compounds **1**–**3**), in contrast to the reference inhibitor Necrostatin-1, which is non-permeant. The cytochrome P450 (CYP) inhibition profile indicates that all three analogues inhibit CYP2C19, CYP2C9, and CYP2D6; CYP1A2 is inhibited by Compounds **1** and **3** (as well as Necrostatin-1), whereas CYP3A4 inhibition is predicted exclusively for Compound **2**. Finally, screening against Pan-Assay Interference Compounds (PAINS) libraries revealed zero alerts for all candidate compounds as well as Necrostatin-1, while synthetic accessibility scores range favorably between 2.94 and 3.14 (compared with 2.58 for the reference scaffold), confirming the chemical tractability and drug-like profile of the examined chemotypes.

**Figure 11:**
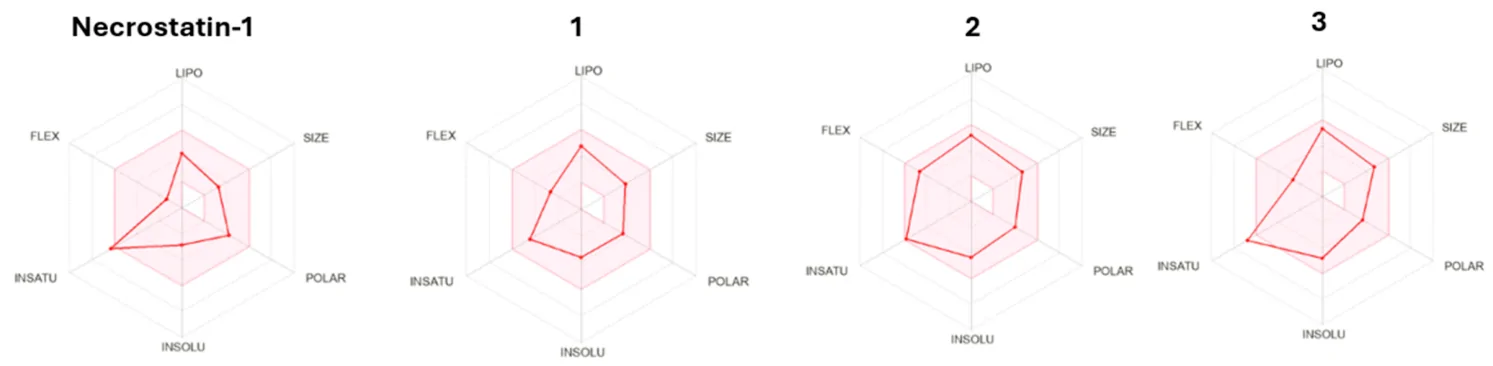
SwissADME bioavailability radar plots for Necrostatin-1 and Compounds **1**, **2**, **3** where the pink shaded zone defines the optimal oral bioavailability space across six physicochemical descriptors.

Predicted oral bioavailability attributes were evaluated using the SwissADME Bioavailability Radar representations (Figure 11), which depict drug-likeness across six core physicochemical properties: lipophilicity (LIPO), molecular size (SIZE), polarity (POLAR), insolubility (INSOLU), unsaturation (INSATU), and flexibility (FLEX). The pink shaded polygon defines the optimal property space for oral drug candidates. The reference inhibitor Necrostatin-1 falls largely within the permissible polygon, displaying slight extension along the unsaturation axis owing to the aromatic indole ring. Compound **1** displays the most favorable alignment among the analogues, falling almost entirely within the permissible boundaries with only a marginal departure along the unsaturation axis due to the aromatic indole ring. Compound **2** displays favorable compliance across size, polarity, solubility, and lipophilicity, showing slight extensions along the flexibility axis and unsaturation axis. Compound **3** exhibits optimal size, polarity, and flexibility, but slightly exceeds the permissible boundary along the unsaturation axis due to the aromatic chlorinated indole and substituted benzyl rings.

## 3. Discussion

The structural targeting of Receptor-Interacting Serine/Threonine-Protein Kinase 1 (RIPK1) represents an established pharmacological strategy to attenuate necroptotic signaling and downstream inflammatory damage. In the present study, a computational workflow comprising virtual screening, induced fit docking, 500 ns molecular dynamics simulations, and in silico pharmacokinetic profiling was implemented to analyze structural analogues of the prototypical inhibitor Necrostatin-1. The structural data derived from the initial docking calculations highlighted that peripheral derivatization on the core hydantoin scaffold significantly influences binding affinity and spatial complementarity within the catalytic cleft (PDB ID: 4ITH). In particular, Compound **3** exhibited superior calculated thermodynamic affinity compared with the reference molecule, which can be attributed to favorable hydrophobic packing coupled with directional cation-pi contacts. Compound **1** similarly demonstrated favorable steric fitting, whereas Compound **2** displayed a lower binding affinity (−4.157 kcal/mol), suggesting that steric hindrance within the binding cleft penalizes the accommodation of the ethoxyphenylethyl group, leading to suboptimal initial placement. The divergent binding orientations reflect steric constraints imposed by the distinct N-substituents: the compact ring of Compound **1** preserves canonical indole alignment and Leu157 hydrogen bonding, whereas the bulky tail of Compound **2** inverts the core toward peripheral residues. Moreover, simulating Compound **2** for 500 ns provided an informative negative control, confirming spontaneous pocket unbinding and validating that unfavorable static scores translate to dynamic instability.

Static docking models, however, are inherently limited by the omission of continuous solvent effects and protein flexibility. The execution of 500 ns Desmond molecular dynamics trajectories provided insight into the conformational endurance of these complexes under dynamic, solvated conditions. While Compound **3** initially displayed the highest docking score (−9.081 kcal/mol), the MD trajectory demonstrated that this initial pose was kinetically unstable, as the ligand drifted by 7–9 Å from its docked coordinates within the first 30 ns. This finding underscores that static scoring alone cannot predict dynamic complex integrity. Nevertheless, Compound **3** did not dissociate completely into the bulk solvent; instead, it re-anchored into a secondary conformation supported by persistent interactions with Asn170, Glu171 and Lys45. In contrast, Compound **1** was the sole candidate that maintained its original crystallographic-like docking pose throughout the entire 500 ns trajectory (RMSD 1.2–2.0 Å) with uninterrupted Leu157 hydrogen bonding, as visually confirmed by the 0 vs. 500 ns structural superimpositions, thereby prioritizing Compound **1** as the primary candidate for synthetic validation.

Complementary in silico pharmacokinetic assessments via SwissADME demonstrated that the investigated compounds possess drug-like physicochemical attributes, with full compliance with Lipinski and Veber criteria and favorable synthetic accessibility scores. The predicted high gastrointestinal absorption and absence of PAINS alerts indicate favorable drug-like physicochemical properties and theoretical permeability. Notably, the predicted blood–brain barrier permeability for Compound **1** represents a promising pharmacokinetic asset, considering the established involvement of RIPK1-driven necroptosis in neurodegenerative disorders and ischemic brain injury, where CNS-penetrant kinase inhibitors are clinically desirable. However, these in silico parameters reflect computed molecular attributes rather than experimental oral bioavailability or in vivo pharmacological viability. Nevertheless, because all findings in this investigation are derived exclusively from computational models, these observations must be treated as preliminary hypotheses. Computational predictions cannot fully account for biological complexity, off-target promiscuity or metabolic clearances in living systems. Consequently, future work will focus on the chemical synthesis of these prioritized derivatives, followed by comprehensive in vitro kinase inhibition assays and subsequent in vivo pharmacokinetic and efficacy evaluations to validate their true biological utility against RIPK1-mediated pathways.

## 4. Materials and Methods

### 4.1. SwissSimilarity Ligand Search

A focused ligand-based virtual screening protocol was conducted using the SwissSimilarity web server (http://www.swisssimilarity.ch, accessed on 5 September 2026) to identify synthesizable structural analogues of the necroptosis inhibitor Necrostatin-1 from the Enamine screening library (Enamine Ltd., Kyiv, Ukraine). The search was executed using the Combined (2D electroshape and 3D shape alignment) approach, which combines 2D fingerprint similarity and 3D shape alignment into a unified consensus score to preserve the central indole-hydantoin pharmacophoric scaffold while sampling chemical diversity around the hydantoin core. Rather than an indiscriminate broad library screening, an analog-based prioritization strategy was applied, and the three top-ranking compounds exhibiting the highest overall similarity scores to Necrostatin-1 were selected from the screening output: Compound **1** (score 0.972), Compound **2** (score 0.965), and Compound **3** (score 0.934). In addition to achieving the highest similarity scores, these prioritized candidates strategically sample three distinct structural modifications at the hydantoin N-position (an aliphatic, sterically constrained ethylcyclobutyl group in Compound **1**; an extended, flexible ethoxyphenylethyl moiety in Compound **2**; and a halogenated, aromatic 3-fluoro-4-methylbenzyl motif in Compound **3**), allowing detailed, computationally intensive evaluation using continuous 500 ns molecular dynamics simulations in explicit solvent [24].

### 4.2. Protein Target Selection and Preparation

The crystallographic structure of the human Receptor-Interacting Serine/Threonine-Protein Kinase 1 (RIPK1) kinase domain was retrieved from the RCSB Protein Data Bank under accession code PDB ID: 4ITH (resolution 2.30 Å). The monomeric Chain A spanning residues Asn17 to Glu299 was selected as the structural model. This crystallographic construct contains four engineered surface-exposed cysteine-to-alanine point mutations (C34A, C127A, C233A, and C240A) introduced to prevent nonspecific aggregation and promote crystallization without perturbing the architecture of the catalytic and allosteric pockets [19]. Target preparation was executed using the Protein Preparation Wizard within the Schrödinger Maestro Suite 2021-2 (release 2021-2, Schrödinger, LLC, New York, NY, USA). The co-crystallized small-molecule ligand (1GA; 7-Cl-Nec-1), co-solvents, and non-structural bulk water molecules distant (>5.0 Å) from the binding pocket were removed, while crystallographic water molecules mediating key hydrogen-bonding interactions within the binding cavity were retained. Bond orders were assigned, hydrogen atoms were added, and amino acid protonation states were optimized at physiological pH 7.4 using PROPKA version v3.x. The hydrogen-bonding network was thoroughly optimized, and the entire system was subsequently relaxed through restrained energy minimization utilizing the OPLS4 force field until the root-mean-square deviation of heavy atoms converged within a threshold of 0.30 Å [25].

### 4.3. Ligand Preparation and Geometry Optimization

Chemical structures of Necrostatin-1 and the three selected putative analogues were prepared within the Maestro environment using LigPrep version 2021-2. Protonation and ionization states were generated at physiological pH 7.4 via Epik version 5.6. Conformation searching and structural geometry refinement were performed with MacroModel version 13.2 using the OPLS4 force field. Energy minimization was executed using a conjugate gradient approach with a strict convergence gradient threshold of 0.01 kcal/mol, ensuring stereochemically and energetically optimized 3D ligand conformations prior to docking calculations [26].

### 4.4. Induced Fit Docking Simulations

Docking calculations were conducted using the Schrödinger Induced Fit Docking (IFD) workflow in Maestro version 2021-2 to account for localized receptor flexibility upon ligand engagement. The receptor grid was centered on the centroid of the co-crystallized inhibitor (7-Cl-Nec-1, Ligand ID: 1GA) within the allosteric pocket of the RIPK1 kinase domain (PDB ID: 4ITH) [19], using an enclosing outer grid box with dimensions of 42 Å × 42 Å × 42 Å to comprehensively encompass the inter-lobe binding cavity and adjacent subpockets, ensuring unconstrained sampling of ligand poses and flexible receptor side chains. In the initial stage of the IFD protocol, softened-potential docking was performed using Glide with van der Waals scaling factors of 0.70 for the receptor and 0.50 for the ligand to accommodate side-chain movement. Residues located within a 5.0 Å radius of each docked ligand pose were subsequently refined and energy-minimized using the Prime module. Following conformational optimization of the active site side chains, ligands were redocked into the relaxed receptor geometries under the Glide Extra Precision (XP) mode. The final ligand poses were ranked based on the comprehensive IFD score. Specific intermolecular interactions, including hydrogen bonds, water-mediated bridges, and cation-pi contacts, were analyzed using the Ligand Interaction Diagram module [27].

To validate the predictive accuracy and structural fidelity of the Induced Fit Docking protocol, self-docking (re-docking) of the co-crystallized ligand (7-Cl-Nec-1, Ligand ID: 1GA) was performed using identical grid parameters and refinement settings. The top-ranked re-docked pose yielded an IFD score of −7.643 kcal/mol and reproduced the native binding mode with a heavy-atom root-mean-square deviation (RMSD) well below the 2.0 Å validation threshold (0.84 Å), fully preserving the anchor hydrogen-bonding interaction with Leu157 and key contacts with Asn170 (Supplementary Materials, Figure S7).

### 4.5. Desmond Molecular Dynamics Simulations

The dynamic stability and conformational evolution of the RIPK1-ligand complexes were investigated by performing 500 ns all-atom molecular dynamics (MD) simulations using the Desmond engine in Schrödinger Suite 2021-2 under the OPLS4 force field [25]. Each docked complex was embedded in an orthorhombic box with explicit TIP3P water molecules, providing a minimum buffer distance of 10.0 Å along all three Cartesian axes (10 × 10 × 10 Å) between the protein surface and box boundaries [28]. The system was electrically neutralized by adding 11 Na^+^ counterions, and a physiological ionic strength of 0.15 M NaCl was established, while excluding ion placement within a 10 Å radius of the bound ligand. Long-range electrostatic interactions were computed using the Particle Mesh Ewald (PME) method, and short-range van der Waals interactions were examined with a cutoff radius of 9.0 Å. Numerical integration was performed using the RESPA integrator with a time step of 2.0 fs for bonded and near non-bonded interactions, and 6.0 fs for far electrostatic interactions. The default Desmond relaxation protocol was applied prior to production. Production simulations were conducted under an isothermal-isobaric (NPT) ensemble for 500 ns at a constant temperature of 310 K controlled by a Nosé–Hoover thermostat and a pressure of 1 bar maintained via the Martyna–Tobias–Klein barostat. Trajectory analyses were focused on calculating the root-mean-square deviation (RMSD) of the protein C-alpha backbone and ligand (fit on protein), as well as evaluating protein–ligand contact persistence and interaction fraction histograms over the entire 500 ns trajectory [28]. All-atom molecular dynamics simulations were performed on a local workstation equipped with an NVIDIA GeForce RTX 3070 GPU accelerator (Asus Dual OC V2, 8 GB GDDR6; NVIDIA Corporation, Santa Clara, CA, USA), utilizing Desmond’s CUDA-enabled implementation for numerical integration.

### 4.6. In Silico Pharmacokinetic Profiling

The pharmacokinetic parameters, physicochemical descriptors, and drug-likeness profiles of the compounds were examined using the SwissADME web server (http://www.swissadme.ch, accessed on 5 September 2026). Examined descriptors included molecular weight, rotatable bonds, topological polar surface area (TPSA), lipophilicity consensus log Po/w, and aqueous solubility estimated via the ESOL model. Gastrointestinal absorption and blood–brain barrier permeation were determined. Furthermore, compounds were assessed for compliance with established medicinal chemistry filters, including Lipinski’s Rule of Five, Veber rules, Pan-Assay Interference Compounds (PAINS) substructural alerts, and synthetic accessibility scores. Bioavailability radar diagrams were constructed to visually analyze property space compliance across lipophilicity, size, polarity, solubility, flexibility, and saturation [29].

## 5. Conclusions

In this study, the binding modes, dynamic stability, and predicted pharmacokinetic properties of three putative Necrostatin-1 analogues were evaluated against the human RIPK1 kinase domain (PDB ID: 4ITH). By employing a computational workflow combining ligand-based virtual screening, induced fit docking, and 500 ns molecular dynamics trajectories, and comparative structural superimpositions, hypotheses regarding putative structural features governing kinase engagement were explored. The static docking simulations indicated distinct energetic rankings, wherein Compound **3** and Compound **1** displayed favorable predicted affinities surpassing the reference inhibitor, while Compound **2** yielded a lower docking score (−4.157 kcal/mol) due to steric constraints within the cleft. The extensive 500 ns Desmond simulations further differentiated these computationally prioritized candidates under solvated conditions, revealing that Compound **1** remained anchored within the primary catalytic cleft with minimal root-mean-square deviations (1.2–2.0 Å) and sustained hydrogen bonds to Leu157. In contrast, Compound **2** exhibited complete displacement from the initial pocket, whereas Compound **3** quickly disengaged from its initial docked conformation (ligand RMSD drifting to 7.0–9.2 Å), indicating that its initial favorable docking score did not translate to dynamic stability, despite establishing a secondary contact network with Asn170, Glu171, and Lys45.

Complementary in silico profiling via the SwissADME platform corroborated that all three derivatives exhibit favorable drug-like properties, characterized by high gastrointestinal absorption, optimal topological polar surface areas, absence of Pan-Assay Interference Compounds alerts, and compliance with Lipinski and Veber filter rules. However, because these conclusions are grounded strictly on in silico methodologies, they represent preliminary structural hypotheses rather than conclusive biological proof. The absence of experimental validation means that potential liabilities such as off-target interactions, cellular toxicity, and in vivo metabolic clearance cannot be definitively excluded. Accordingly, future studies will concentrate on the synthetic execution and biological validation of the prioritized lead candidate Compound **1**, followed by comprehensive in vitro kinase inhibition panels, cellular necroptosis assays and subsequent in vivo preclinical evaluations to definitively ascertain its therapeutic efficacy as a RIPK1 modulator.

## Figures and Tables

**Table 1:** Induced Fit Docking scores and structural descriptions for Necrostatin-1 and the three derivatives docked into RIPK1 (PDB ID: 4ITH).

Compound	Chemical Structure	Docking Score (kcal/mol)
Necrostatin-1	* 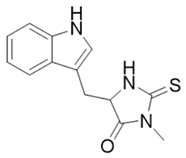 *	−7.337
Compound **1**	* 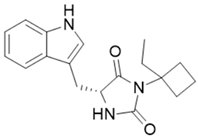 *	−7.793
Compound **2**	* 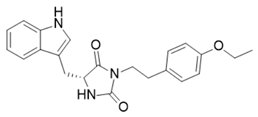 *	−4.157
Compound **3**	* 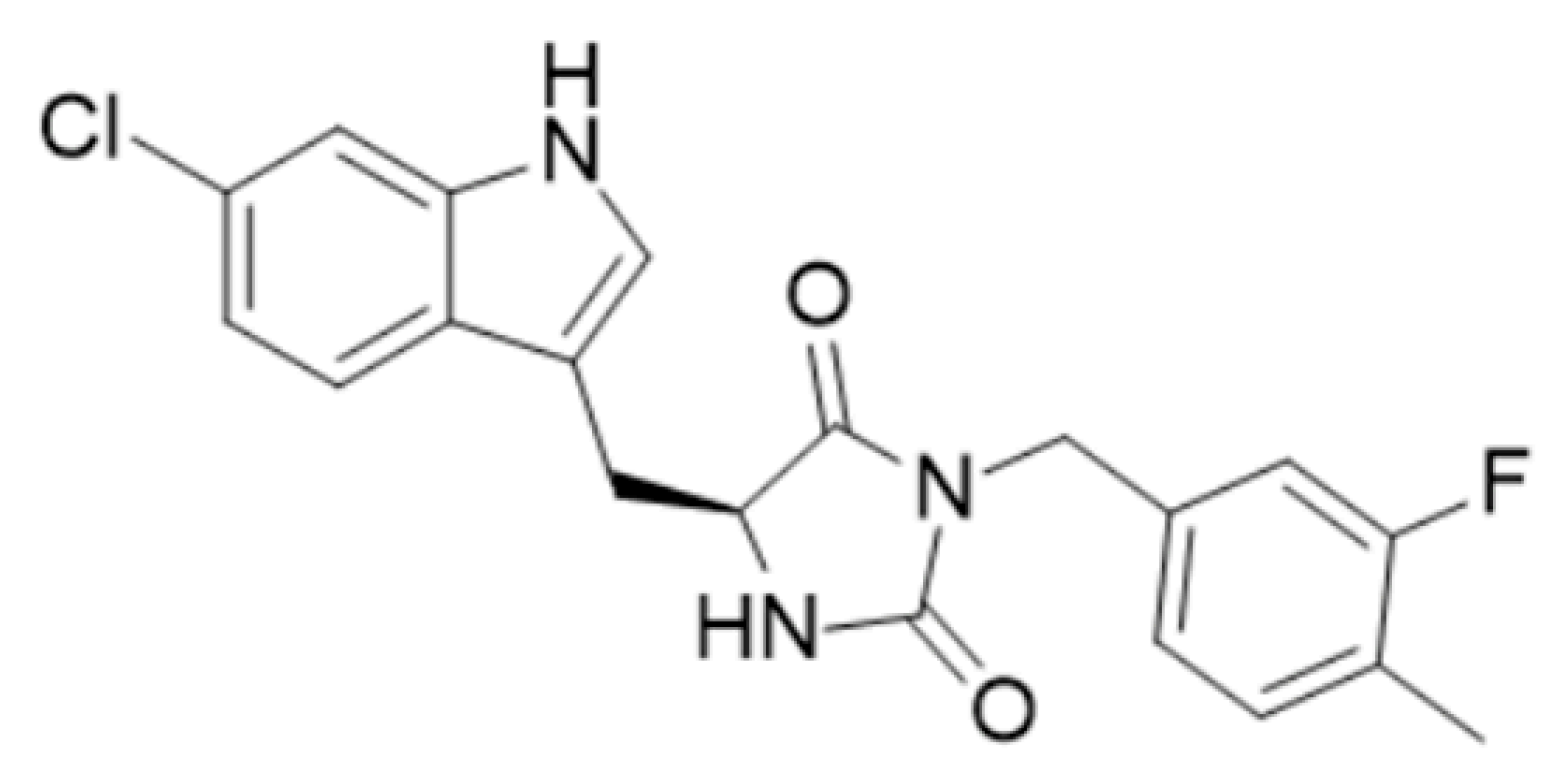 *	−9.081

**Table 2:** In silico pharmacokinetic, physicochemical, and medicinal chemistry properties of Necrostatin-1 and Compounds **1**, **2**, **3** calculated via SwissADME. HBA and HBD definitions follow the standard SwissADME conventions (HBA: sum of N and O atoms; HBD: sum of OH and NH groups).

Parameter	Necrostatin-1	Compound 1	Compound 2	Compound 3
Molecular Weight (g/mol)	259.33	311.38	377.44	385.82
Heavy Atoms	18	23	28	27
Aromatic Heavy Atoms	9	9	15	15
Fraction Csp3	0.23	0.44	0.27	0.20
Rotatable Bonds	2	4	7	4
H-bond Acceptors	1	2	3	3
H-bond Donors	2	2	2	2
Molar Refractivity	81.90	96.65	115.29	109.12
TPSA (Å^2^)	80.22	65.20	74.43	65.20
Consensus Log Po/w	1.51	2.52	2.91	3.36
ESOL Log S (Class)	−2.86 (Soluble)	−3.61 (Soluble)	−4.35 (Moderately soluble)	−4.80 (Moderately soluble)
GI Absorption	High	High	High	High
BBB Permeant	No	Yes	Yes	Yes
P-gp Substrate	No	Yes	No	No
CYP1A2 Inhibitor	Yes	Yes	No	Yes
CYP2C19 Inhibitor	Yes	Yes	Yes	Yes
CYP2C9 Inhibitor	No	Yes	Yes	Yes
CYP2D6 Inhibitor	No	Yes	Yes	Yes
CYP3A4 Inhibitor	No	No	Yes	No
Log Kp (Skin permeation, cm/s)	−6.56	−6.15	−6.08	−5.93
Lipinski Violations	0	0	0	0
Veber Violations	0	0	0	0
Bioavailability Score	0.55	0.55	0.55	0.55
PAINS Alerts	0	0	0	0
Synthetic Accessibility	2.58	2.94	3.14	3.01

## Data Availability

The original contributions presented in this study are included in the article. Further inquiries can be directed to the corresponding author.

## Supplementary Materials

The supporting information can be downloaded at https://www.mdpi.com/article/10.3390/ijms27219616/s1.
