# Supplementary material for: Computational Evaluation of Necrostatin-1 Analogues as Potential RIPK1 Inhibitors
Source: Int J Mol Sci. 2026 Oct 27;27(21):9616. doi: 10.3390/ijms27219616 (PMC13763241; doi:10.3390/ijms27219616)
Supplement: Supplementary file 1 [file ijms-27-09616-s001.zip › ijms-4637370-supplementary.pdf]

Supplementary Materials

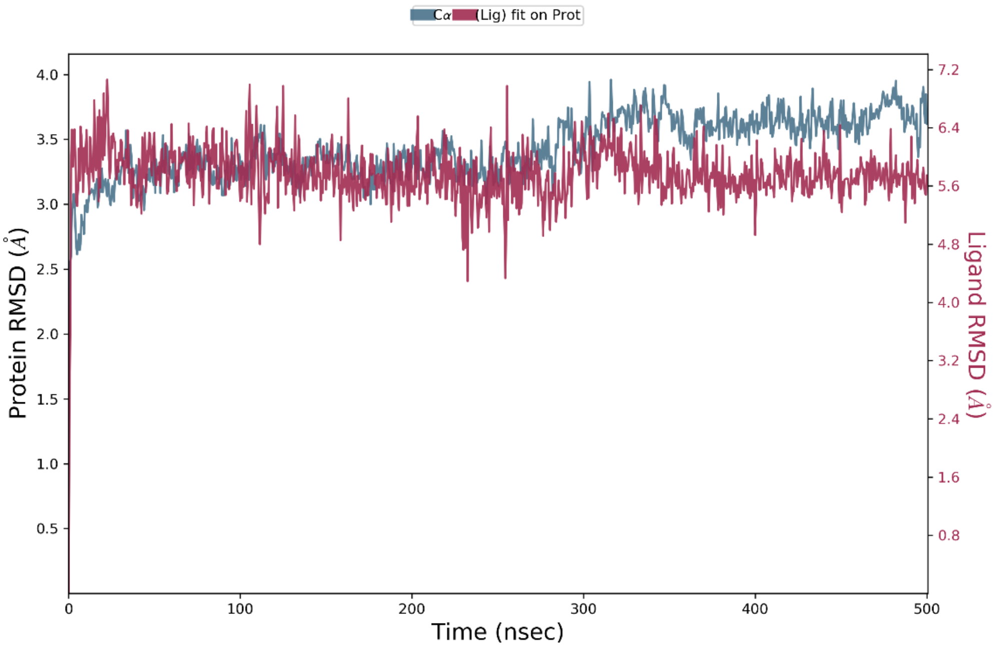

Figure S1. Root-mean-square deviation (RMSD) trajectory of Necrostatin-1 in complex with RIPK1 over 500 ns.

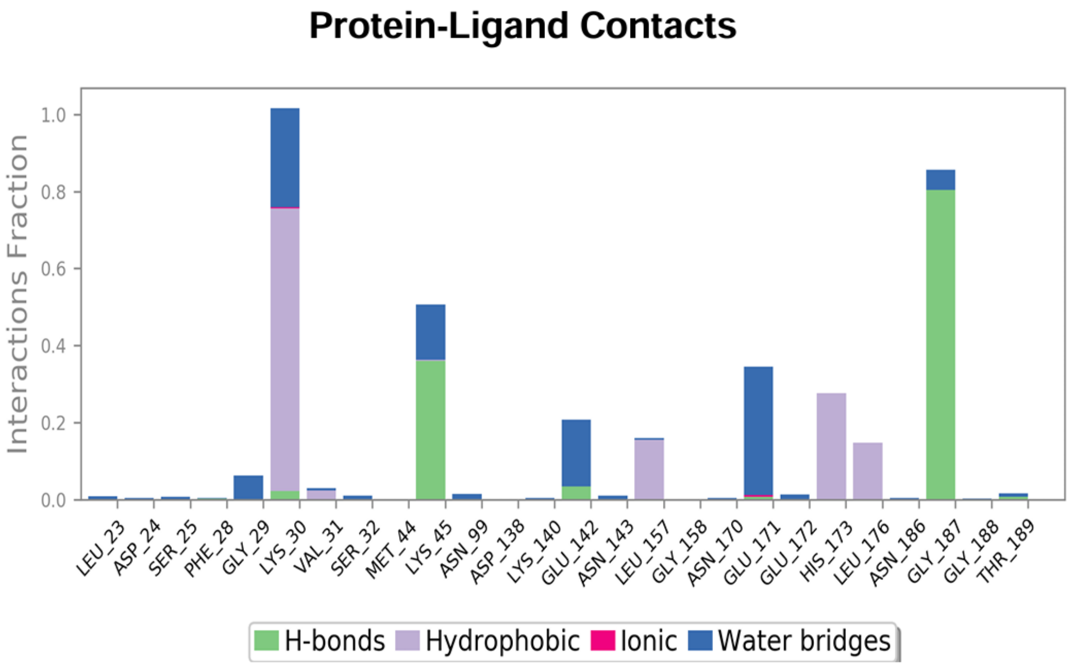

Figure S2. Protein–ligand contacts histogram for the reference inhibitor Necrostatin-1 in complex with the RIPK1 kinase domain (PDB ID: 4ITH) across the 500 ns molecular dynamics trajectory, depicting interaction fractions categorized into hydrogen bonds, hydrophobic contacts, and water bridges.

Molecule 1

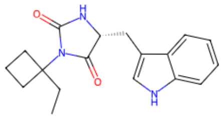

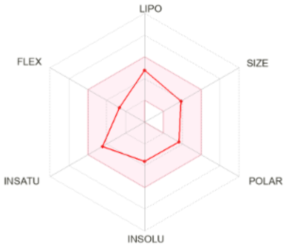

SMILES CCC1(CCC1)N1C(=O)N[C@@H](C1=O)Cc1c[nH]c2c1cccc2

Physicochemical Properties	
Formula	C18H21N3O2
Molecular weight	311.38 g/mol
Num. heavy atoms	23
Num. arom. heavy atoms	9
Fraction Csp3	0.44
Num. rotatable bonds	4
Num. H-bond acceptors	2
Num. H-bond donors	2
Molar Refractivity	96.65
TPSA	65.20 Å²

Lipophilicity	
Log $P_{o/w}$ (iLOGP1.5)	3.17
Log $P_{o/w}$ (XLOGP3)	2.88
Log $P_{o/w}$ (WLOGP)	2.20
Log $P_{o/w}$ (MLOGP)	1.85
Log $P_{o/w}$ (SILICOS-IT)	2.50
Consensus Log $P_{o/w}$	2.52

Water Solubility	
Log *S* (ESOL)	-3.61
Solubility	7.63e-02 mg/ml ; 2.45e-04 mol/l
Class	Soluble
Log *S* (Ali)	-3.91
Solubility	3.84e-02 mg/ml ; 1.23e-04 mol/l
Class	Soluble
Log *S* (SILICOS-IT)	-3.50
Solubility	9.85e-02 mg/ml ; 3.16e-04 mol/l
Class	Soluble

Pharmacokinetics	
GI absorption	High
BBB permeant	Yes
P-gp substrate	Yes
CYP1A2 inhibitor	Yes
CYP2C19 inhibitor	Yes
CYP2C9 inhibitor	Yes
CYP2D6 inhibitor	Yes
CYP3A4 inhibitor	No
Log K_p (skin permeation)	-6.15 cm/s

Druglikeness	
Lipinski	Yes; 0 violation
Ghose	Yes
Veber	Yes
Egan	Yes
Muegge	Yes
Bioavailability Score	0.55

Medicinal Chemistry	
PAINS	0 alert
Brenk	1 alert: hydantoin
Leadlikeness	Yes
Synthetic accessibility	2.94

Figure S3. SwissADME pharmacokinetic prediction of Compound 1.

Molecule 1

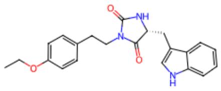

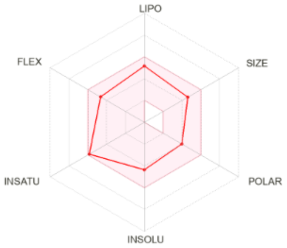

SMILES CCOc1ccc(cc1)CCN1C(=O)N[C@@H](C1=O)Cc1c[nH]c2c1cccc2

Physicochemical Properties	
Formula	C22H23N3O3
Molecular weight	377.44 g/mol
Num. heavy atoms	28
Num. arom. heavy atoms	15
Fraction Csp3	0.27
Num. rotatable bonds	7
Num. H-bond acceptors	3
Num. H-bond donors	2
Molar Refractivity	115.29
TPSA	74.43 Å²

Lipophilicity	
Log $P_{o/w}$ (iLOGP1.5)	3.31
Log $P_{o/w}$ (XLOGP3)	3.55
Log $P_{o/w}$ (WLOGP)	2.51
Log $P_{o/w}$ (MLOGP)	1.98
Log $P_{o/w}$ (SILICOS-IT)	3.20
Consensus Log $P_{o/w}$	2.91

Water Solubility	
Log *S* (ESOL)	-4.35
Solubility	1.68e-02 mg/ml ; 4.46e-05 mol/l
Class	Moderately soluble
Log *S* (Ali)	-4.80
Solubility	6.01e-03 mg/ml ; 1.59e-05 mol/l
Class	Moderately soluble
Log *S* (SILICOS-IT)	-5.42
Solubility	1.42e-03 mg/ml ; 3.77e-06 mol/l
Class	Moderately soluble

Pharmacokinetics	
GI absorption	High
BBB permeant	Yes
P-gp substrate	No
CYP1A2 inhibitor	No
CYP2C19 inhibitor	Yes
CYP2C9 inhibitor	Yes
CYP2D6 inhibitor	Yes
CYP3A4 inhibitor	Yes
Log K_p (skin permeation)	-6.08 cm/s

Druglikeness	
Lipinski	Yes; 0 violation
Ghose	Yes
Veber	Yes
Egan	Yes
Muegge	Yes
Bioavailability Score	0.55

Medicinal Chemistry	
PAINS	0 alert
Brenk	1 alert: hydantoin
Leadlikeness	No; 2 violations: MW>350, XLOGP3>3.5
Synthetic accessibility	3.14

Figure S4. SwissADME pharmacokinetic prediction of Compound 2.

Molecule 1

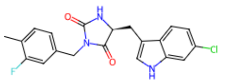

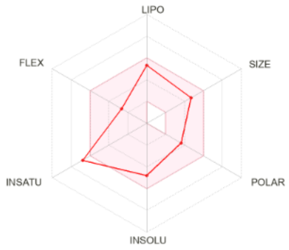

SMILES Clc1ccc2c(c1)[nH]cc2C[C@@H]1NC(=O)N(C1=O)Cc1ccc(c(c1)F)C

Physicochemical Properties	
Formula	C20H17ClFN3O2
Molecular weight	385.82 g/mol
Num. heavy atoms	27
Num. arom. heavy atoms	15
Fraction Csp3	0.20
Num. rotatable bonds	4
Num. H-bond acceptors	3
Num. H-bond donors	2
Molar Refractivity	109.12
TPSA	65.20 Å²
**Lipophilicity**	
Log $P_{o/w}$ (iLOGP1.5)	3.04
Log $P_{o/w}$ (XLOGP3)	3.84
Log $P_{o/w}$ (WLOGP)	3.44
Log $P_{o/w}$ (MLOGP)	2.95
Log $P_{o/w}$ (SILICOS-IT)	3.53
Consensus Log $P_{o/w}$	3.36

Water Solubility	
Log *S* (ESOL)	-4.80
Solubility	6.14e-03 mg/ml ; 1.59e-05 mol/l
Class	Moderately soluble
Log *S* (Ali)	-4.91
Solubility	4.80e-03 mg/ml ; 1.24e-05 mol/l
Class	Moderately soluble
Log *S* (SILICOS-IT)	-5.53
Solubility	1.13e-03 mg/ml ; 2.94e-06 mol/l
Class	Moderately soluble
**Pharmacokinetics**	
GI absorption	High
BBB permeant	Yes
P-gp substrate	No
CYP1A2 inhibitor	Yes
CYP2C19 inhibitor	Yes
CYP2C9 inhibitor	Yes
CYP2D6 inhibitor	Yes
CYP3A4 inhibitor	No
Log K_p (skin permeation)	-5.93 cm/s
**Druglikeness**	
Lipinski	Yes; 0 violation
Ghose	Yes
Veber	Yes
Egan	Yes
Muegge	Yes
Bioavailability Score	0.55
**Medicinal Chemistry**	
PAINS	0 alert
Brenk	1 alert: hydantoin
Leadlikeness	No; 2 violations: MW>350, XLOGP3>3.5
Synthetic accessibility	3.01

Figure S5. SwissADME pharmacokinetic prediction of Compound 3.

Molecule 1

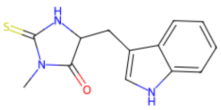

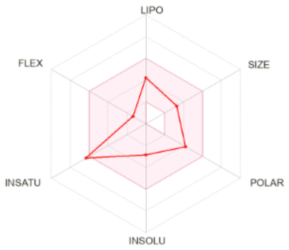

SMILES S=C1NC(C(=O)N1C)Cc1c[nH]c2c1cccc2

Physicochemical Properties	
Formula	C13H13N3OS
Molecular weight	259.33 g/mol
Num. heavy atoms	18
Num. arom. heavy atoms	9
Fraction Csp3	0.23
Num. rotatable bonds	2
Num. H-bond acceptors	1
Num. H-bond donors	2
Molar Refractivity	81.90
TPSA	80.22 Å²
**Lipophilicity**	
Log $P_{o/w}$ (iLOGP1.5)	1.98
Log $P_{o/w}$ (XLOGP3)	1.86
Log $P_{o/w}$ (WLOGP)	0.66
Log $P_{o/w}$ (MLOGP)	0.62
Log $P_{o/w}$ (SILICOS-IT)	2.42
Consensus Log $P_{o/w}$	1.51

Water Solubility	
Log *S* (ESOL)	-2.86
Solubility	3.60e-01 mg/ml ; 1.39e-03 mol/l
Class	Soluble
Log *S* (Ali)	-3.17
Solubility	1.77e-01 mg/ml ; 6.82e-04 mol/l
Class	Soluble
Log *S* (SILICOS-IT)	-2.22
Solubility	1.55e+00 mg/ml ; 5.97e-03 mol/l
Class	Soluble
**Pharmacokinetics**	
GI absorption	High
BBB permeant	No
P-gp substrate	No
CYP1A2 inhibitor	Yes
CYP2C19 inhibitor	Yes
CYP2C9 inhibitor	No
CYP2D6 inhibitor	No
CYP3A4 inhibitor	No
Log K_p (skin permeation)	-6.56 cm/s
**Druglikeness**	
Lipinski	Yes; 0 violation
Ghose	Yes
Veber	Yes
Egan	Yes
Muegge	Yes
Bioavailability Score	0.55
**Medicinal Chemistry**	
PAINS	0 alert
Brenk	1 alert: thiocarbonyl_group
Leadlikeness	Yes
Synthetic accessibility	2.58

Figure S6. SwissADME pharmacokinetic prediction of Necrostatin-1.

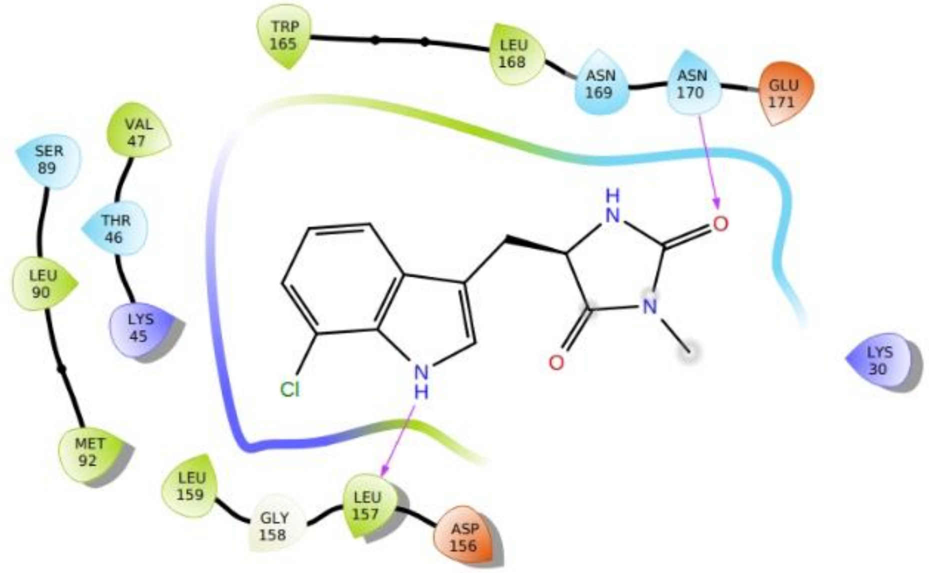

Figure S7. Two-dimensional ligand interaction diagram of the re-docked co-crystallized inhibitor (7-Cl-Nec-1, Ligand ID: 1GA) within the RIPK1 kinase domain (PDB ID: 4ITH; docking score: −7.643 kcal/mol). The docked pose preserves key intermolecular contacts, including the anchor hydrogen bond between the indole NH and the backbone carbonyl of Leu157, alongside polar interactions with Asn170 and surrounding hydrophobic packing.
